# Biomass-Derived Porous Carbon from Agar as an Anode Material for Lithium-Ion Batteries

**DOI:** 10.3390/nano12010022

**Published:** 2021-12-22

**Authors:** Nurbolat Issatayev, Gulnur Kalimuldina, Arailym Nurpeissova, Zhumabay Bakenov

**Affiliations:** 1 National Laboratory Astana, Nazarbayev University, Kabanbay Batyr Ave. 53, Nur-Sultan 010000, Kazakhstan; nurbolat.issatayev@nu.edu.kz (N.I.); arailym.nurpeissova@nu.edu.kz (A.N.); 2 Department of Chemical and Materials Engineering, School of Engineering and Digital Sciences, Nazarbayev University, Kabanbay Batyr Ave. 53, Nur-Sultan 010000, Kazakhstan; 3 Department of Mechanical and Aerospace Engineering, School of Engineering and Digital Sciences, Nazarbayev University, Kabanbay Batyr Ave. 53, Nur-Sultan 010000, Kazakhstan

**Keywords:** activated carbon, agar, anode, lithium-ion battery, chemical activators

## Abstract

New porous activated carbons with a high surface area as an anode material for lithium-ion batteries (LIBs) were synthesized by a one-step, sustainable, and environmentally friendly method. Four chemical activators—H_2_SO_4_, H_3_PO_4_, KOH, and ZnCl_2_—have been investigated as facilitators of the formation of the porous structure of activated carbon (AC) from an agar precursor. The study of the materials by Brunauer–Emmett–Teller (BET) and scanning electron microscopy (SEM) methods revealed its highly porous meso- and macro-structure. Among the used chemical activators, the AC prepared with the addition of KOH demonstrated the best electrochemical performance upon its reaction with lithium metal. The initial discharge capacity reached 931 mAh g^−1^ and a reversible capacity of 320 mAh g^−1^ was maintained over 100 cycles at 0.1 C. High rate cycling tests up to 10 C demonstrated stable cycling performance of the AC from agar.

## 1. Introduction

Lithium-ion batteries (LIBs) are conquering the worldwide market due to their high specific capacity, absence of memory effect, long service life, and stable operation [1,2,3,4,5]. LIBs are extensively used in portable electronics applications and are currently expanding into the field of plug-in hybrid and electric vehicles [6]. Thus, the requirements for LIBs are increasing not only in terms of specific energy of its components (cathode, anode) but also in their eco-friendliness owing to growing awareness of environmental issues. Therefore, the development of electrode materials that correspond to the stated requirements is in high demand.

Nowadays, in commercially available LIBs, graphite is mainly used as an anode material. Despite this fact, some critical shortcomings are associated with graphite—such as a low specific capacity, poor rate capability, and slow charge–discharge processes—limiting their applicability in the next generation technologies in electronics and electric transport. Furthermore, it is worth mentioning the high cost and negative environmental impact due to its synthesis from fossil carbon sources such as coal and petroleum coke [7]. Therefore, it is highly desirable to explore alternative negative electrode materials for Li-ions such as tin [8,9], transition metal oxides [10,11,12,13], silicon [14,15], and others. Although the alternative anodes can provide higher specific capacity, they suffer from substantial volume changes during insertion/extraction of Li-ions causing materials disintegration leading to huge irreversible capacity loss and poor cyclability. The challenges faced by non-carbon anode materials hinder their broad application in the next-generation LIBs. Hence, considerable research efforts continue on the development of carbonaceous materials for high-capacity anodes, especially for the porous carbon materials with the high surface area were extensively researched due to their enhanced electrochemical performance in reaction with Li-ions [16]. This improvement has been associated with porous structure that forms shorter distances for Li-ions and electrons to travel, facilitating enhanced charge transfer, diffusion rate, and better kinetics [17].

Various types of biomass have been intensively researched as a promising renewable source to prepare porous and functional carbon materials. Their low cost, tunable physical/chemical properties, recyclability, abundant availability, and harmony with the environment are especially of high interest [18]. Two mechanisms of Li-ion storage in carbonaceous materials derived from biomass can be defined as the invertible lithiation/delithiation into/out of the expanded graphitic layers and the surface-induced capacitive behavior summoned by pores, regions of structural defects, and functional groups [19]. Pursuant to earlier research, biomass activated carbon nanostructures can be produced and generated from a variety of natural resources such as rice husk [20], wheat stalk [21], cellulose [22], green tea wastes [23], natural cotton [24], puffed corn [25], sucrose [26].

Physical activation [27], chemical activation [28], catalytic activation [29], and microwave activation [30] are commonly used methods for producing biomass-derived AC materials. Among them, chemical activation is extensively utilized to produce porous carbon due to its advantages over other activation methods—such as high yield, less surface damage, low activation temperature, and short reaction time. During the activation process, an activator is usually added to form a new porous structure to increase the specific surface area of the material. The most common chemical activators are Bronsted (H_2_SO_4_, H_3_PO_4_) [31], Lewis acids (CuCl_2_ and ZnCl_2_) [32], alkali metal hydroxides, and carbonates (NaOH, KOH, K_2_CO_3_, Na_2_CO_3_) [33,34,35].

In this work, we propose a facile method of preparation of AC material with a large surface area and a developed porous structure from agar. Agar biopolymer was used as a carbon precursor due to its low cost, high carbon content, and the lack of any traces of heavy metals. The pore formation and expanding effect of chemical activators as H_2_SO_4_, H_3_PO_4_, KOH, and ZnCl_2_ on the AC morphology were compared and studied. The materials were examined as an anode for LIBs and demonstrated high capacity and excellent cycling and rate characteristics. To the best of our knowledge, this is the first application of agar activated carbon as anode for LIBs. Previous studies of activated carbon with agar precursor have been investigated for supercapacitors [36,37]. In addition, the activation of agar at the molecular level was performed according to the method described by Chen et al. [36], which requires a one-step calcination of the agar without high-temperature carbonation.

## 2. Materials and Methods

Agar, KOH, H_3_PO_4_, H_2_SO_4_, ZnCl_2_, and HCl were purchased from Sigma-Aldrich (St. Louis, MO, Germany). All the reagents were analytical grade and used as received without further purifications.

### 2.1. Fabrication of the Activated Carbon

**Scheme 1:**
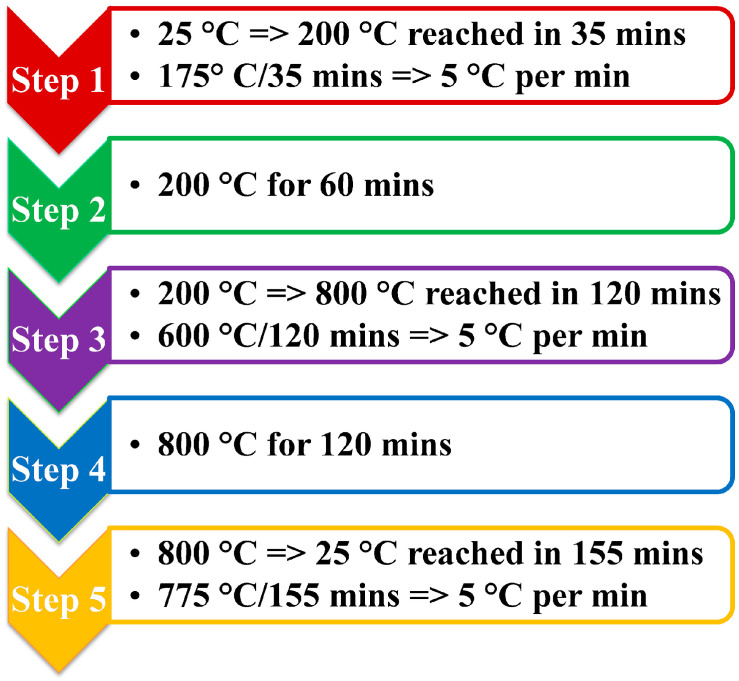
Optimal temperature and time for preparation of AC from agar.

**Figure 1:**
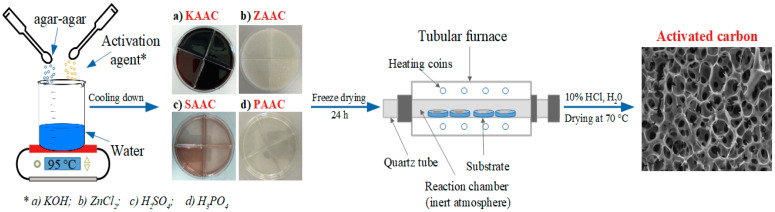
Preparation method of AC with four different activating agents: (**a**) KOH; (**b**) ZnCl_2_; (**c**) H_2_SO_4_; (**d**) H_3_PO_4_.

For preparing the porous activated carbon material, 0.25 g of each KOH, H_3_PO_4_, H_2_SO_4_, and ZnCl_2_ was mixed with agar separately in 10 mL water (DI) at 95 °C. The mixture was stirred until homogeneous hydrogels were obtained. After cooling to room temperature, the mixture was further freeze-dried for 24 h. The sample was pyrolyzed at 800 °C (the heat treatment steps are shown schematically in Scheme 1) in a conventional furnace for 2 h in argon. Finally, after rinsing with 10% HCl and an ample amount of water, the activated carbon was dried at 70 °C in a vacuum oven for 24 h. The preparation route is illustrated in Figure 1. The final agar-derived activated by KOH, H_3_PO_4_, H_2_SO_4_, and ZnCl_2_ carbons were denoted as KAAC, PAAC, SAAC, and ZAAC, respectively.

### 2.2. Characterizations

The resulting AC powders were analyzed by Raman spectroscopy (Horiba LabRam Evolution) with a 633 nm excitation laser range from 200 to 2000 cm^−1^. The functional groups of the ACs were examined using Nicolet iS10 Fourier Transform Infrared (FTIR) Spectrometer in the range of 400–4000 cm^−1^ and X-ray photoelectron spectrometer (XPS)-NEXSA (Thermo Scientific, Waltham, MA, USA). The morphology of the disordered carbons was observed by scanning electron microscope Crossbeam 540 (SEM, Zeiss, Germany) and transmission electron microscope JEOL JEM—1400 Plus (TEM, JEOL, Peabody, MA, USA). Thermogravimetric analysis (TGA) was carried out by Simultaneous Thermal Analyzer (STA, PerkinElmer, Waltham, MA, USA) 6000 at a heating rate of 10 °C min^−1^ from 30 to 800 °C in nitrogen (N_2_). The surface area and pore size distribution of the powders were studied by N_2_ adsorption on Autosorb iQ and ASiQwin gas sorption system (Quantachrome instruments; Boynton Beach, FL, USA).

### 2.3. Cell Preparation

The electrochemical activity of the carbon samples was examined using CR2032-type coin cells. The electrodes were prepared by mixing the obtained ACs, carbon black (Super-P), and polyvinylidene fluoride (PVDF) in the ratio of 8:1:1 in N-methyl-2-pyrrolidone (NMP). The resulting mixture was then applied onto copper foil using the Doctor Blade technique and then dried overnight at 60 °C in a vacuum oven. The coated copper foil was cut into the disks with a diameter of 14 mm. The mass loadings of the active materials were approximately 1–1.5 mg cm^−2^. The cells were assembled in an argon-filled glove box (MBRAUN, LABmaster Pro Glovebox, Germany, containing <0.1 ppm O_2_ and <0.1 ppm H_2_O) using lithium metal foil as the counter and reference electrode. A 1 M LiPF_6_ in ethylene carbonate (EC) and dimethyl carbonate (DMC) (1:1, v/v) was used as an electrolyte solution and a polypropylene membrane (Celgard^®^ 2400) as a separator. Each cell contains~150 μL of electrolyte. Galvanostatic cycling of the electrodes was investigated by a multichannel battery testing system (Arbin Inc. and Neware Battery tester, Neware Co., Shenzhen, China) between the cut-off potentials of 0.01 and 3.0 V vs. Li^+^/Li at a current density of 0.1 and 1.0 C for 100 cycles. Cyclic voltammetry (CV) measurements were performed using a VMP3 potentiostat/galvanostat (Bio-Logic Science Instrument Co.) in a potential range of 0.01–3.0 V at a scan rate of 0.1 mV s^−1^.

## 3. Results and Discussion

**Figure 2:**
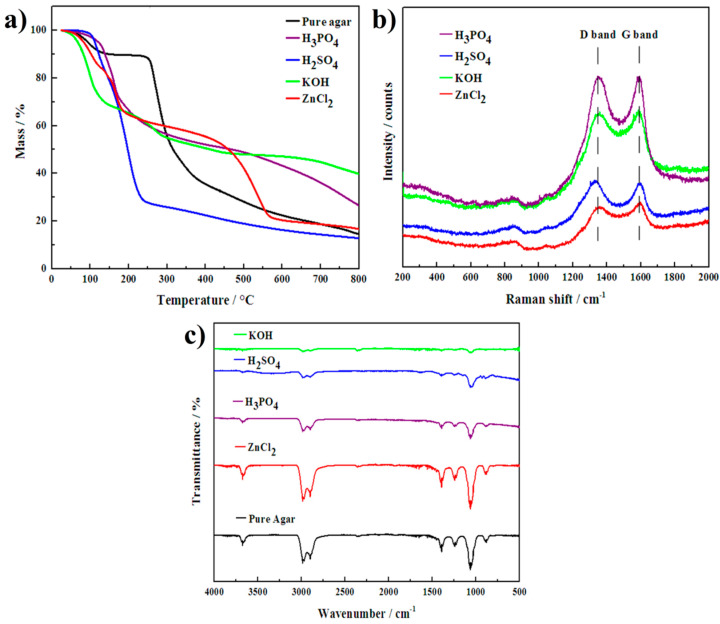
(**a**) TGA curves of pure agar and hydrogels with different activating agents; (**b**) Raman spectra of obtained carbons; (**c**) FT-IR spectra of pure agar and obtained carbons.

The TGA curves (Figure 2a) for all samples, including pure agar, consist of two distinct zones. At the first step, the weight loss of around 5–10% was registered at the temperatures around 50–130 °C which can be associated with water loss. Then there is an abrupt transition at about 210 °C, indicating that the decomposition process involves rapid weight loss. At the temperature of 800 °C, the weight loss reaches approximately 85.5%, 87.2%, 83.3%, 73.4%, and 60.2% for pure agar, SAAC, ZAAC, PAAC, and KAAC, respectively. Comparison of the obtained activated carbons illustrates that KOH activation results in a lower peak temperature, shorter pyrolysis duration, and thermal decomposition at a higher instantaneous rate. These results also imply that the activation of KOH and H_3_PO_4_ is faster and easier than the activation of H_2_SO_4_ and ZnCl_2_.

The structural characteristics of obtained AC were investigated by Raman spectroscopy, as illustrated in Figure 2b. The patterns of all four samples showed two distinct peaks for the G band at about 1580 cm^−1^ assigned to carbon atoms with the sp^2^ electronic configuration in the structure of the graphite sheet, and the D band at about 1340 cm^−1^, which corresponds to the presence of a disordered and defective structure of carbon materials. The degree of disorder in carbon can be evaluated from the ratio of the integrated intensities of the D-band to the G-band (I_D_/I_G_), and higher values represent a greater number of imperfection in carbon atoms. The I_D_/I_G_ ratios of KAAC, PAAC, SAAC, and ZAAC are 1.02, 0.99, 0.98, and 0.97, respectively. It can be seen that the ratio of I_D_/I_G_> 1 for the KOH sample, which emphasizes the largest number of defects and boundaries in the resulting sp^2^-hybridized graphene sheet. A decrease in the ratio for the other three samples (<1) indicates a decrease in the number of voids or defects and, therefore, an upper degree of graphitization [38]. However, all four samples illustrate the high-intensity ratio, which indicates a high amorphous degree, edges, and other defects.

Furthermore, the structural characteristics of the materials were studied by FT-IR spectroscopy. As shown in Figure 2c, all obtained ACs retain some functional group of agar. From the FT-IR spectra, one can observe vibrational peaks at approximately 3650 cm^−1^ which illustrates the -OH (hydroxyl) group and at around 2905 cm^−1^ attributed to the -CH_3_O- (methoxyl) group. In addition, the presence of the peaks at about 933 cm^−1^ and 1075 cm^−1^ are related to the 3,6-anhydrogalactose bridges. However, some peaks of the obtained ACs are less intense. This can be explained by the cleavage of some functional groups during activation [39]. For example, in the process of activation with KOH, it was suggested that the cyclic ether bond of six-membered rings and hydroxyl groups with hydrogen bonds in agar undergoes a dehydration reaction with potassium hydroxide, which is clearly seen from a decrease in the intensity of broad absorption, the band at 3650 cm^−1^ and the peak at 1075 cm^−1^ [40,41]. Analysis of the XPS spectra on the ACs C1s confirmed the partial graphitization and the presence of oxygen functional groups on the surface and is in good agreement with the results of Raman and FT-IR spectroscopy (Figure S1).

**Figure 3:**
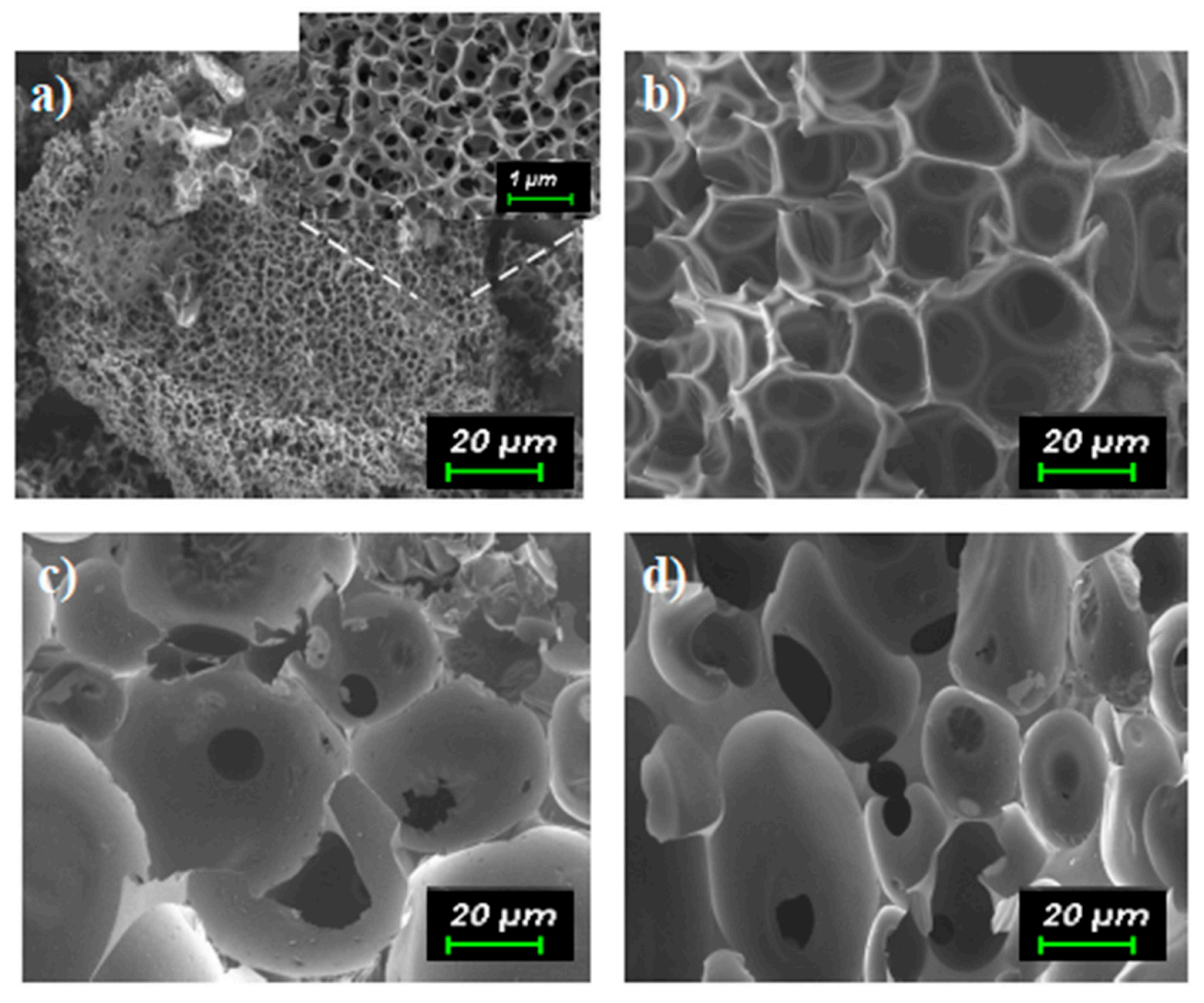
SEM images of ACs with four different activating agents: (**a**) KAAC; (**b**) ZAAC; (**c**) SAAC; (**d**) PAAC.

The morphological characteristics of ACs were studied using SEM as shown in Figure 3. All samples have a porous three-dimensional (3D) structure, which facilitates the better access of the electrolyte to the active material and accelerates the migration of electrons and ions when the materials are used in LIBs. KAAC and ZAAC have a similar 3D interconnected hierarchical structure with meso- and microporous morphology as illustrated in Figure 3a,b. Acid-ACs have similar 3D structures with mesopores (Figure 3c,d). However, the pore sizes of the resulting materials differ, which is discussed further below. Further TEM analysis was performed for the ACs. A disordered hierarchical porous structure can be observed that contains meso- and micro-pores (see Figure S2). The presence of abundant pores in the ACs from the agar can be identified by the large number of white spots between the disordered carbon layers [42].

**Figure 4:**
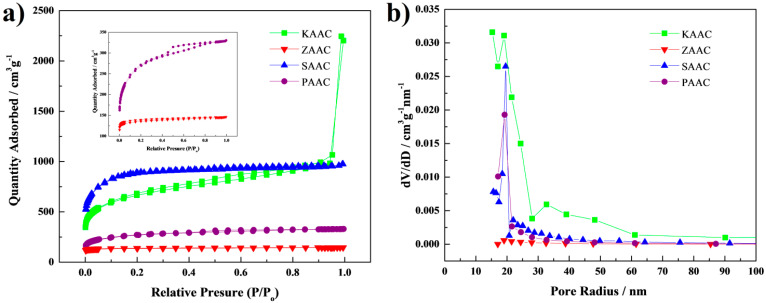
(**a**) Nitrogen adsorption–desorption isotherms and (**b**) pore sizes distribution.

The detailed studies of the porous structure and surface of ACs were carried out by nitrogen adsorption measurements. As shown in Figure 4a, all samples demonstrate a type IV desorption isotherm. When relative pressure is above 0.8, the isotherm increases significantly due to capillary condensation. The desorption isotherm is situated over the adsorption isotherm, which means that the samples have a high amount of mesopores [43]. In Figure 4b, which shows pore size distribution diagrams, it can be seen that the pore size ranges from 12 to 60 nm. The specific surface areas for KAAC, ZAAC, PAAC, and SAAC were defined as 2417.5, 3309.0, 974.1, and 548.3 m^2^ g^−1^, while the average pore size was 15.2, 19.5, 19.2, and 19.0 nm, respectively. The results indicate that the acid activators and ZnCl_2_ tend to form a mesoporous structure, while using a KOH activator results in meso- and macroporous structures.

**Figure 5:**
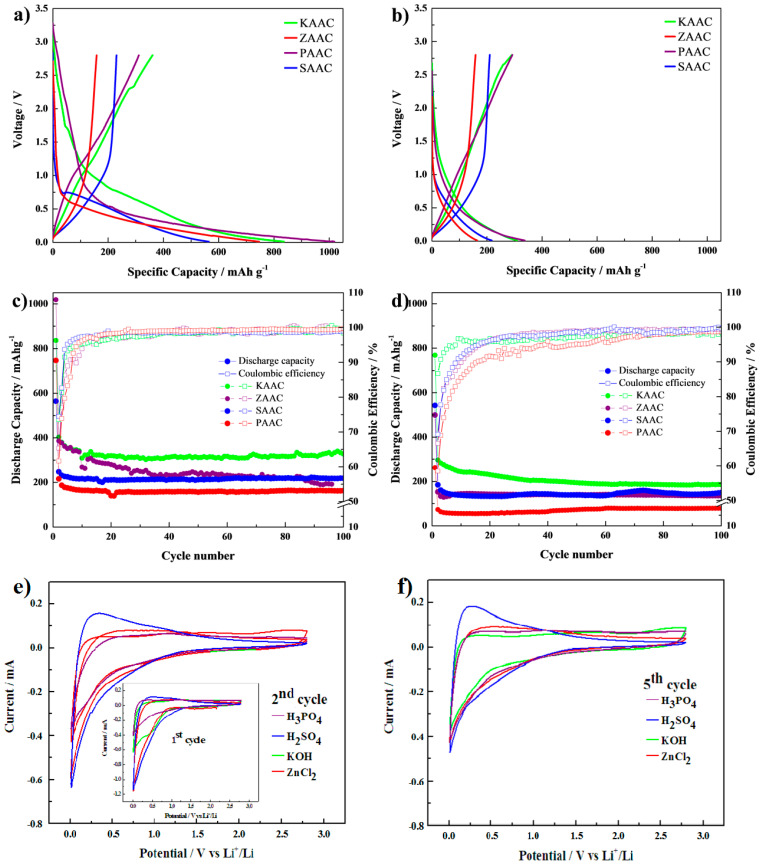
Cycling performances of AC electrodes (**a**) at 1st cycle at 0.1 C rate; (**b**) at 10th cycle at 0.1 C rate. Cycling performance with Coulombic efficiency (right *Y*-axis) of PAAC, SAAC, KAAC, and ZAAC cycled at (**c**) 0.1 C rate; (**d**) 1 C rate. CV profiles of PAAC, SAAC, KAAC, and ZAAC at a scan rate of 0.1 mV s^−1^, from 0.01 to 2.8 V: (**e**) 2nd cycle and (**f**) 5th cycle.

The prepared ACs were tested as anode materials in LIBs. Figure 5a,b show the charge–discharge profiles of the electrodes at a charge–discharge rate of 0.1 C. At the initial cycle, KAAC, PAAC, SAAC, and ZAAC exhibited charge/discharge capacities of 360/836, 311/1018, 230/564, and 110/597 mAh g^−1^, respectively. The Coulombic efficiencies were 42.9%, 30.5%, 40.8% and 18.4% for KAAC, PAAC, SAAC, and ZAAC, respectively. The low Coulombic efficiency in the 1st cycle is caused by the decomposition of the electrolyte, the formation of an SEI layer, and some irreversible capture of Li ions in a special position of the carbonaceous material, which may be associated with a surface reaction, for example, near residual H atoms [44]. At the 10th cycle, the charge-specific capacities for KAAC, PAAC, SAAC, and ZAAC were 290, 292, 169, and 123 mAh g^−1^, while the discharge-specific capacities were 309, 337, 174, and 132 mAh g^−1^, respectively. The Coulombic efficiency improved significantly in the 10th cycle and amounted to 95, 88, 97, and 96%, respectively. The increase in the Coulombic efficiency might be in the formation of a stabilized SEI film. The SEI layer acts as a passivation layer, the formation of which is advantageous for carbon anodes as it prevents the continuous electrolyte decomposition on the activated carbon electrode, and therefore the anode is in a stable state [7].

The CV curves of the samples ranging from 0.01 to 2.8 V (versus Li/Li^+^) at a scan rate of 0.1 mV s^−1^ are illustrated in Figure 5e,f. It is clearly seen that the 1st cycle differs from the second and fifth cycles. The area of the reduction peak of the 1st cycle is more significant than that of the second and fifth cycles. This difference is associated with the non-reversible intercalation of Li-ions into the ACs [45]. There are two peaks for KAAC at around 0.01–0.2 V and 0.45 V at the first cycle. The latter peak corresponds to the decomposition and generation of SEI on the surface of carbon that disappears in subsequent cycles owing to the stabilization of the SEI layer. The influence of the SEI layer is vital for the CV cycle and Coulombic efficiency [46]. Consequently, at the first cycle, the Coulomb efficiency was low as shown in Figure 5, but in further cycles, it stabilized. A peak around 0.01 V is related to the incorporation of Li-ions into the layered carbon, and a flat peak between 0.2 and 0.6 V is attributed to the extracting process of Li-ions from the electrode [47]. The CV curves of the second and fifth cycles have a partial overlap, which indicates the outstanding stability of the electrode material.

To investigate the cyclic stability of the prepared carbons, the cells were galvanostatically cycled at current densities of 0.1 C and 1 C for 100 cycles as shown in Figure 5c,d. The stable discharge capacity of KAAC was the highest reversible capacity at 0.1 and 1 C compared to PAAC, SAAC, and ZAAC. The specific capacity for KAAC, PAAC, SAAC, and ZAAC was 330, 239, 212, and 157 mAh g^−1^ at 0.1 A g^−1^ and 190, 150, 136, and 79 mAh g^−1^ at 1 C, respectively. The reversible capacity of KAAC is higher than recently reported ACs obtained from cherry pit activated with KOH and H_3_PO_4_ [48]. Comparison with other bio-derived activated carbons is illustrated in Table 1. Although upon the initial cycles the Coulombic efficiency of all samples was low, it increased upon further cycling and reached almost 100%. These results demonstrate the excellent cycling capability and reversibility of all samples. Such tendencies can be observed practically for bio-mass derived activated carbon used as anode materials [7,48,49].

To determine the structural stability of the activated carbon anodes, the electrodes were analyzed by SEM before and after 20 cycles. SEM images show that the electrodes before and after cycles have a rough surface with many pores, which indicates the stability of the electrodes (Figure S3). In order to further explain the reversible surface redox reaction at the porous anode, the CVs of the SAAC anode were performed at various scan rates (Figure S4a). The linkage between peak currents and scan rates, which is almost linear with a correlation of 0.999, was studied using redox peaks of roughly 0.64 V (Figure S4b). This result shows that the redox reaction is surface-limited rather than diffusion-limited [50]. As indicated earlier, porous carbon materials have a large surface area. As follows, the quantity of oxygen-containing functional groups and other functional groups on the surface of carbon corresponds to the surface area of the porous material. The beforementioned oxygen-containing functional groups participate in the redox reaction with lithium-ion, schematically described as: C=O + Li^+^ + e^−^ => C-O-Li. Consequently, the pseudo-capacitive effect, prompted by surface reactions, has a significant effect on the advancement of electrochemical energy storage. Moreover, this effect might explain the high specific capacity and excellent performance of the activated porous carbon materials in lithium-ion batteries [51].

**Figure 6:**
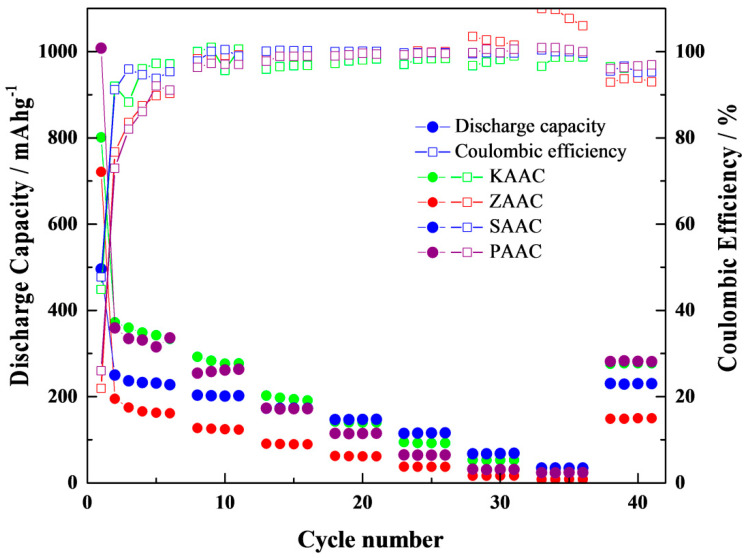
Rate capability of AC electrodes with ZnCl_2_, H_2_SO_4_, KOH, and H_3_PO_4_ activators from 0.1 to 10 C.

Furthermore, the rate performance of samples was investigated by charging and discharging the cells at various current densities between 0.1 and 10 C each for 5 cycles as illustrated in Figure 6. At a current density of 0.1 C, the specific discharge capacities of KAAC, PAAC, SAAC, and ZAAC were 359, 331, 232, and 161 mAh g^−1^, respectively. Although the specific capacity of the samples reduced upon increasing the cycling current density to 0.5, 1, 2, 5, and 10 C, the cells could recover their original value when the current density was changed back to 0.1 C. The pore system and large S_BET_ of the samples are likely to facilitate the rapid transfer of Li-ions and enhance the rate characteristics.

## 4. Conclusions

In summary, we have successfully prepared low-cost and sustainable ACs using a renewable agar precursor activated by four different activators. Through investigating the effect of the activators on the morphology of AC, it was found that the AC anode obtained with KOH as an activating agent achieved the best electrochemical performance. The initial discharge capacity of AC with KOH reached 931 mAh g^−1^ and a reversible capacity of 326 mAh g^−1^ was delivered over 100 cycles at 0.1 C. The Coulombic efficiency remained above 96% after 10 cycles. Furthermore, all prepared samples exhibited excellent capacity retention and reversibility upon prolonged cycling and high rate capability. This research demonstrates a simple and effective method for preparation of mesoporous AC, and provides an effective synthesis route for a production of unique 3D templates that can be used to host various electrode materials.

## Figures and Tables

**Table 1:** Comparison of the electrochemical characteristics of different bio-derived ACs used as anode materials for lithium-ion batteries (LIBs)

AC Precursor	Activating Agent	Measurement Conditions	Initial Capacity (mAh g^−1^)	Reversible Capacity (mAh g^−1^)	References
Agar	KOH	0.1 C	931	320 at 0.1 C after 100 cycles	This work
Sisal fiber	KOH	0.1 C	646	Capacity loss of 363 compared to initial capacity	[5]
Corn straw	CaCl_2_	0.2 C	1534	546 at 0.2 after 100 cycles	[7]
Shaddock peel	KOH	50 mAg^−1^	1284	673 at t 50 mAg^−1^ after 100 cycles	[19]
Wheat stalk	KOH	0.1 C	502	139.6 at 10 C after 3000 cycles	[21]
Green tea wastes	KOH	0.1 A/g	706	400 at 0.1 A/g after 100 cycles	[23]
Jute fiber	ZnCl_2_	0.2 C	2117	742.7 at 0.2 C after 100 cycles	[32]
Pomelo peels	-	90 mAg^−1^	756	452 at 90 mAg^−1^ after 200 cycles	[44]
Bamboo chopsticks	KOH	∼0.37 C	∼500	∼360 at ∼0.37 C after 100 cycles	[45]
Cherry pit	KOH	C/3	-	210 at C/3 after 200 cycles	[48]

## Data Availability

The data presented in this study are available on request from the corresponding author.

## Supplementary Materials

The following are available online at https://www.mdpi.com/article/10.3390/nano12010022/s1, Figure S1: XPS spectra of (a) KAAC; (b) ZAAC; (c) PAAC; (d) SAAC, Figure S2: TEM images of ACs with four different activating agents: (a) SAAC; (b) PAAC; (c) KAAC; (d) ZAAC, Figure S3: SEM images of pristine electrodes: (a) KAAC, (c) PAAC, (e) ZAAC, and (g) SAAC, and electrodes after 20 cycles: (b) KAAC, (d) PAAC, (f) ZAAC, and (h) SAAC, Figure S4: (a) CV curves of SAAC electrodes at various scan rates of 1.0 mV s^−1^, 1.5 mV s^−1^, and 2.0 mV s^−1^; (b) Relationship between the redox peak current and scanning rates.
