# Supplementary material for: Biomass-Derived Porous Carbon from Agar as an Anode Material for Lithium-Ion Batteries
Source: Nanomaterials (Basel). 2021 Dec 22;12(1):22. doi: 10.3390/nano12010022 (PMC8746817; doi:10.3390/nano12010022)
Supplement: Supplementary file 1 [file nanomaterials-12-00022-s001.zip › nanomaterials-1478354-supplementary.pdf]

nanomaterials

Supplementary materials

Biomass-Derived Porous Carbon from Agar as an Anode Material for Lithium-ion Batteries

Nurbolat Issatayev [1,2] Gulnur Kalimuldina [3,*], Arailym Nurpeissova[1], and Zhumabay Bakenov [1,2,*]

[1] National Laboratory Astana, Nazarbayev University, Kabanbay Batyr Ave. 53, Nur-Sultan 010000, Kazakhstan; nurbolat.issatayev@nu.edu.kz (N.I.); arailym.nurpeissova@nu.edu.kz (A.N.); zbakenov@nu.edu.kz (Z.B.)
[2] Department of Chemical and Materials Engineering, School of Engineering and Digital Sciences, Nazarbayev University, Kabanbay Batyr Ave. 53, Nur-Sultan 010000, Kazakhstan;
[3] Department of Mechanical and Aerospace Engineering, School of Engineering and Digital Sciences, Nazarbayev University, Kabanbay Batyr Ave. 53, Nur-Sultan 010000, Kazakhstan; gkalimuldina@nu.edu.kz
* Correspondence: authors: gkalimuldina@nu.edu.kz ; Tel.: +7-717-269-4578, zbakenov@nu.edu.kz; Tel: +7-717-270-65-30

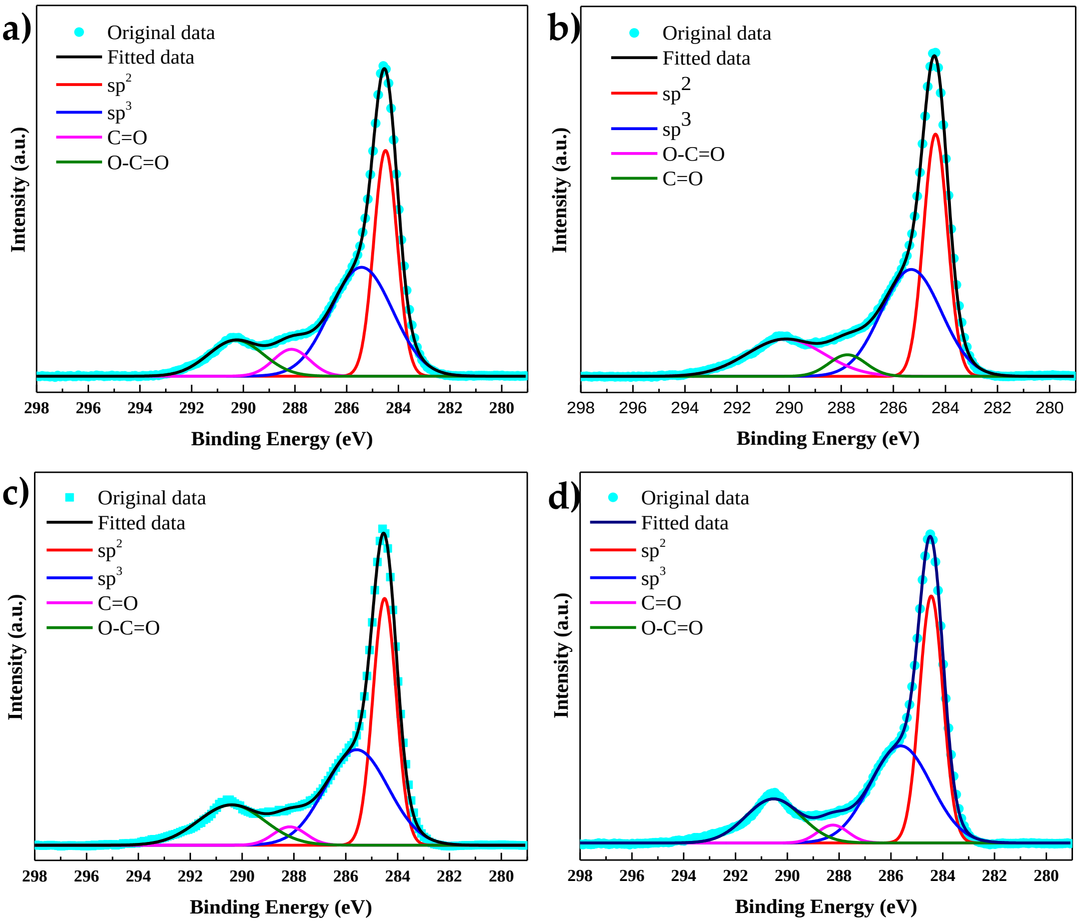

Figure S1. XPS spectra of **a**) KAAC; **b**) ZAAC; **c**) PAAC; **d**) SAAC.

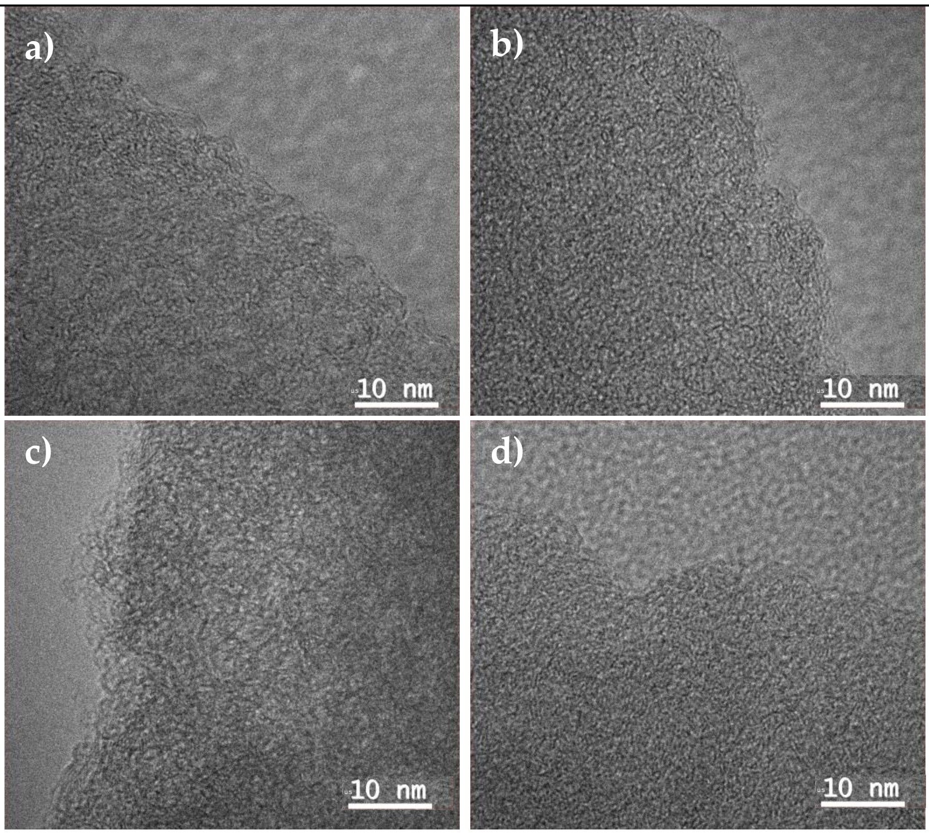

Figure S2. TEM images of ACs with four different activating agents: **a**) SAAC**;** **b**) PAAC; **c**) KAAC; **d**) ZAAC.

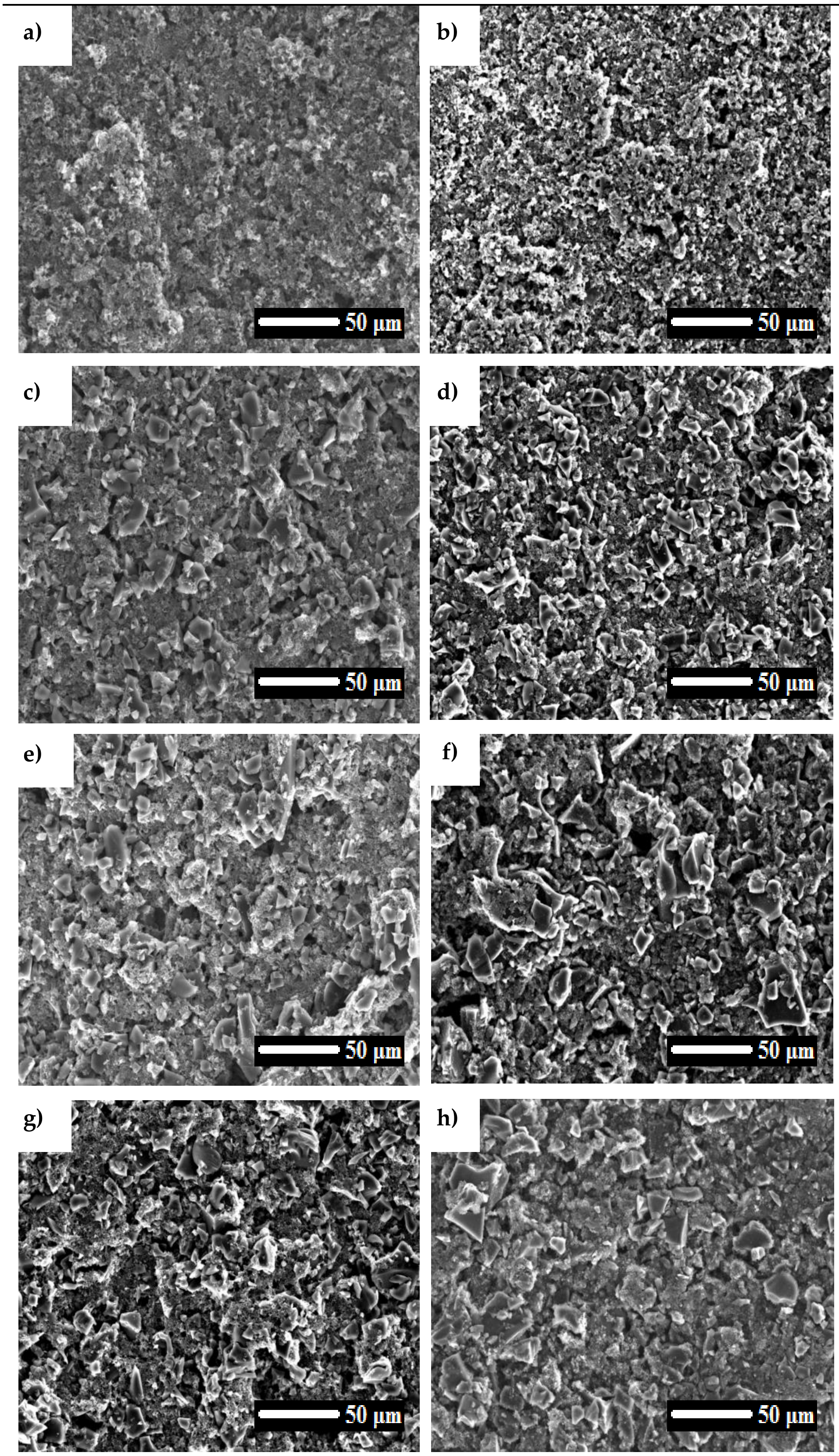

Figure S3. SEM images of pristine electrodes: **a**) KAAC, **c**) PAAC, **e**) ZAAC, and **g**) SAAC, and electrodes after 20 cycles: **b**) KAAC, **d**) PAAC, **f**) ZAAC, and **h**) SAAC.

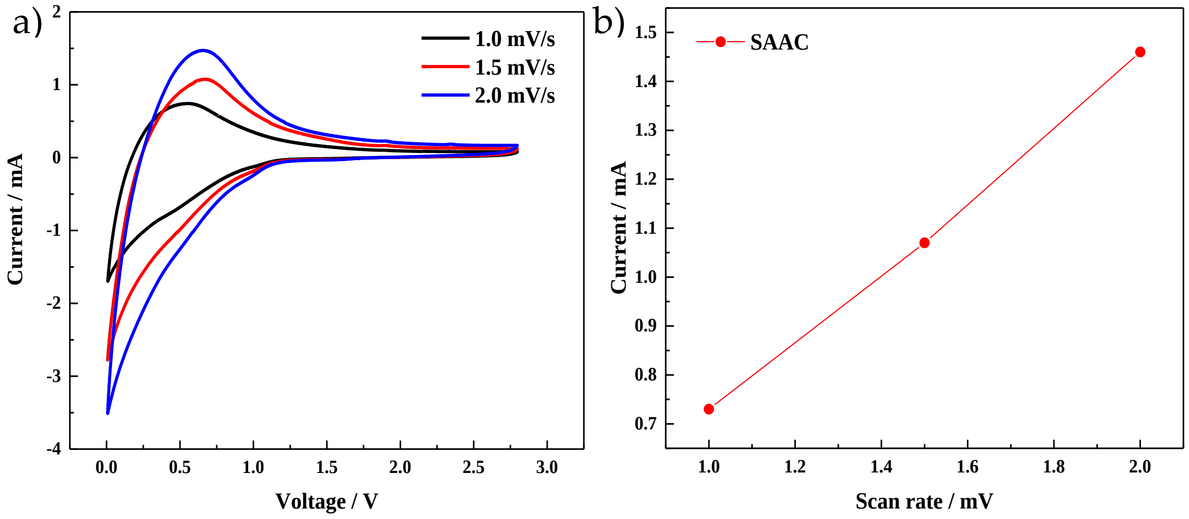

Figure S4. a) CV curves of SAAC electrodes at various scan rates of 1.0 mV s^{-1}, 1.5 mV s^{-1}, and 2.0 mV s^{-1}; **b**) Relationship between the redox peak current and scanning rates.
